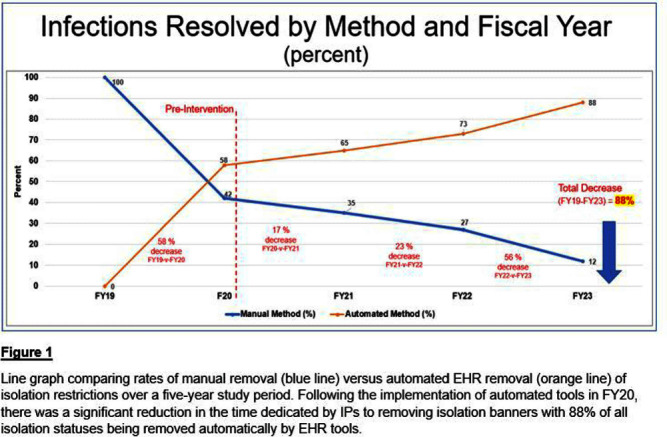# Automated Discontinuation of Isolation Precautions with the Use of Electronic Health Record Tools

**DOI:** 10.1017/ash.2024.304

**Published:** 2024-09-16

**Authors:** Sherry Cantu, Amy Spallone, Roy Chemaly, Jane Powell, Ha Bui, Nadeem Prasla

**Affiliations:** MD Anderson Cancer Center; The University of Texas MD Anderson Cancer Center

## Abstract

**Background:** At a comprehensive cancer center, hundreds of patients are screened daily for infections requiring the implementation of isolation precautions. Discontinuation of precautions is determined by negative testing, resolution of infection, or other criteria. Determining appropriate discontinuation of isolation precautions is labor intensive for Infection Preventionists (IPs). An unintended consequence of manual discontinuation is that numerous patients remain on isolation indefinitely. This was amplified during the COVID-19 pandemic when thousands of patients were placed on isolation precautions. Using electronic health record (EHR) tools, opportunities for process improvements were developed. Our goal was to establish an automated method to resolve isolation precautions. We aimed to decrease the number of manually resolved precautions by 25% each fiscal year (FY), compared to our baseline of activity in FY 2019 (FY19). Our secondary aim was to automate adding and resolving precautions when testing is initiated for suspected transmissible conditions (rule-out testing features).

**Methods:** Infection Control (IC) collaborated with EHR analysts to build tools to automate a process for appropriate isolation discontinuation. We reviewed our internal data in conjunction with evidence-based guidelines and started with acute, short-term infections that do not require repeat testing or cultures. Expiration dates were established for these infections to resolve automatically after meeting criteria. A secondary review determined that additional infections could be added safely to this process. The secondary aim of establishing rule-out testing was implemented for respiratory viral panels (including SARS-COV-2) and C. difficile testing. When testing was ordered for these conditions, a suspect-infection status and alert for precautions were automatically added to patients’ EHR banners. If the assay resulted negative, the suspect-infection status was automatically removed from their chart. **Results:** Our baseline of active infections in FY19 was approximately 2,700 cases. From FY19 through FY23, 123,115 infections were added to our patients, and 128,422 infections were resolved. In the first year of implementation, there was a 58% decrease in the number of manually resolved cases. From the initiation of our project through the end of FY23, manual discontinuation of precautions has decreased by 88%. **Conclusions:** We successfully implemented a process improvement project to appropriately remove patients from isolation precautions automatically using EHR tools, which resulted in reduced labor on our IPs and patient time spent on isolation restrictions. Additional benefits from this process improvement extend to decreasing unnecessary costs to the patient and the organization, better stewardship of supply/resources, and improving patient satisfaction.

**Figure:**